# Iron Oxide–Chitosan Macroporous Nanocomposite Hydrogels for Efficient Heterogeneous Electro-Fenton Degradation of Ciprofloxacin

**DOI:** 10.3390/gels12050434

**Published:** 2026-05-15

**Authors:** José Benito Pelayo-Vázquez, Daryl Rafael Osuna-Laveaga, José Patricio Peña-Jaramillo, Sergio Gómez-Salazar, Edgar David Moreno-Medrano, María Guadalupe Pérez-García

**Affiliations:** 1 Centro Universitario de Tonalá, Universidad de Guadalajara, Tonalá 45425, Jalisco, Mexico; benito.pelayo@academicos.udg.mx (J.B.P.-V.); daryl.osuna@academicos.udg.mx (D.R.O.-L.); jpcenerin@gmail.com (J.P.P.-J.); 2 Centro Universitario de Ciencias Exactas e Ingenierías, Universidad de Guadalajara, Guadalajara 44430, Jalisco, Mexico; sergio.gsalazar@academicos.udg.mx

**Keywords:** electro-Fenton, ciprofloxacin, iron oxide–chitosan hydrogels, Pickering HIPEs, heterogeneous catalysis, wastewater treatment, sustainable materials

## Abstract

Ciprofloxacin (CIP) is a persistent fluoroquinolone antibiotic frequently detected in water bodies, and its efficient mineralization remains a challenge in wastewater treatment. In this work, iron oxide–chitosan macroporous nanocomposite hydrogels were developed as heterogeneous catalysts for the electro-Fenton degradation of CIP. The materials were synthesized via Pickering high internal phase emulsion templating, yielding monoliths with a three-dimensional interconnected porous structure, an average pore size of 18.9 ± 0.7 µm, a window size of 8.1 ± 0.7 µm, an openness degree of 39.6%, a specific surface area of 1.77 m^2^ g^−1^, an iron content of 64.2 mg g^−1^, and a crosslinking degree of 92.1%. The monoliths exhibited controlled swelling in aqueous medium at pH 3, with a gravimetric water uptake of 142.1 ± 2.3% and a volumetric swelling of 39.3 ± 1.2% at equilibrium. Iron oxide particles remained exposed on the porous surface, providing accessible catalytic sites, while the interconnected porosity favored reactant diffusion. Compared with direct anodic oxidation, which achieved 32% total organic carbon removal after 20 min, the heterogeneous electro-Fenton process using the synthesized monoliths as catalysts showed superior performance, reaching nearly 95% removal within 2 min and complete mineralization within 15 min. This enhanced performance was associated with higher hydroxyl radical generation (~3.5 µM) than that observed for anodic oxidation alone (~1.5 µM). These findings highlight the potential of biodegradable iron oxide–chitosan macroporous hydrogels as sustainable catalysts for antibiotic removal from water.

## 1. Introduction

Antibiotics are antibacterial agents that have been structurally designed to maximize their biological activity at low doses and to produce a prolonged action on living beings [1]. These drugs are widely used for humans and animals to prevent or treat diseases caused by bacterial infections [1,2]. Antibiotics can hardly be decomposed thoroughly in a living body by physiological activities, so they are commonly excreted into the environment. They can enter the aquatic systems as micropollutants and cause human health and aquatic life problems [1,2,3]. For instance, ciprofloxacin (CIP) is a synthetic antibiotic drug of second-generation fluoroquinolones that has been found in surface water and even in ground and drinking water [4,5]. As a result, CIP has induced acute and sub-lethal effects on most species of freshwater consumers. In addition, Ebert et al. reported that CIP poses a risk to photoautotrophic organisms and is toxic to cyanobacteria even at low concentrations (e.g., µgL^−1^) [5]. Therefore, there is great interest in developing effective techniques for the complete removal of antibiotics and their metabolites to avoid contamination of water bodies.

Advanced oxidation processes (AOPs) have shown great potential for the removal of organic pollutants (including antibiotics) [6,7] in wastewater treatment. AOPs generate highly effective reactive oxygen species (such as hydroxyl radicals, •OH) during the reaction process that convert most organic pollutants to smaller compounds and even to CO_2_ [7,8,9,10]. Among these methods, the Fenton process is one of the most cost-effective AOPs. In this process, the organic pollutants are easily degraded by •OH and hydroperoxyl radicals (•OOH/O_2_) that are generated by the reaction between Fe^2+^ ions and H_2_O_2_ in the bulk solution [7,8,9,10,11]. Even so, the homogeneous Fenton reaction presents some drawbacks, mainly due to the large consumption of H_2_O_2_, the reaction is carried out at acid conditions (pH < 3), the excessive formation of ferric hydroxide sludge, and the loss of the catalyst in the effluent [8,10,11]. To overcome these disadvantages, some researchers have gradually begun to pay attention to the heterogeneous electro-Fenton process [10]. For this purpose, Fe ions have been immobilized on several solid support materials (e.g., activated carbon [12,13], biochar [14,15], clays [16], polymers [17,18,19], etc.) with different structural configurations, which avoids the costly Fe separation and the ferric hydroxide sludge formation as happens in a conventional homogeneous Fenton process. In addition, H_2_O_2_ is instantly electrogenerated in situ by the reduction of e^-^ oxygen in the cathode (which acts as an acceptor), reducing the risks derived from handling this compound [10,18]. The electric field allows the rapid generation of •OH radicals, as a result of the reaction between the hydrogen peroxide electrogenerated and the iron-supported catalyst, which improves the degradation of organic pollutants [10,18].

Chitosan (CS), a linear natural polysaccharide obtained from the deacetylation of chitin, has been widely recognized as an efficient and sustainable support for iron-based catalysts due to its low toxicity, low cost, abundance, biodegradability, and strong metal-binding capability [18,20,21]. The biodegradable nature of chitosan is particularly important, as it enables the safe disposal of the material at the end of its service life without generating secondary environmental contamination. In addition, the incorporation of a well-defined porous structure can further enhance catalytic performance by improving the accessibility of reactive species to the active sites and facilitating mass transport within the material [11,17,20,21]. However, to the best of our knowledge, macroporous chitosan-based materials have been scarcely investigated as supports for heterogeneous electro-Fenton catalysis [20,21]. This gap highlights the need to develop structurally tailored, biodegradable catalytic systems that combine high accessibility of active sites with enhanced mass transport properties.

In this regard, the high internal phase emulsion (HIPE) templating technique has been widely used for the synthesis of polymers with well-defined and controllable porous structures [22,23]. HIPEs are formulated with a high volume fraction of internal (higher than the maximum packing volume fraction 74%) within a polymerizable continuous phase. The structure of HIPEs consists of polyhedral droplets separated by a thin film of the continuous phase. The polymerization of the continuous phase and the subsequent removal of the internal one allow the creation of interconnected 3D macroporous materials (called in several works as polyHIPEs) that are structural replicas of the precursor emulsions [22,24]. HIPEs can be stabilized by surfactants [22,24], particles (nano or microscale) [25,26] or surfactant/particles hybrids [27,28,29,30]. If particles are used as stabilizer agents, emulsions are termed Pickering HIPEs. The porous structure and thus the specific surface area of polyHIPEs can be easily tailored by modifying the type or concentration of surfactant and/or nanoparticles, the internal to continuous phase volume ratio, the viscosity of the internal phase, etc. [28,29,30,31]. In the case of Pickering HIPEs, the porous surface can be easily functionalized because (nano or micro) particles are embedded onto the porous surface after polymerization and purification, which allows to obtain polyHIPE nanocomposites for several applications, including water treatment [27,28,29,30], tissue engineering [32], electricity generation [33], among others. In water treatment, several polyHIPEs have been extensively used as adsorbents/absorbents of different pollutants, including metal ions [34,35], dyes [36], oils [29,37], drugs [38], etc.). However, there are few reports of polyHIPEs used as iron-supported catalysts for the Fenton process despite the advantages that the HIPE method offers in this matter [28,39]. Magnetite (Fe_3_O_4_) nanoparticles (NPs) are particularly attractive as iron catalysts due to their ability to facilitate the Fe^2+^/Fe^3+^ redox cycle, which is essential for the continuous activation of H_2_O_2_ and the generation of hydroxyl radicals (•OH), as well as their good chemical stability, low toxicity, and high availability [40]. For instance, Zhang et al. [39] synthesized magnetic polyacrylamide macroporous beads by sedimentation polymerization of pristine Fe_3_O_4_ NPs stabilized oil-in-water HIPEs. These researchers reported that the polyHIPE beads proved to be an excellent reusable catalyst of the conventional Fenton reaction for decomposition of the methylene orange dye due to the presence of Fe_3_O_4_ NPs onto the porous surface and the open porous structure.

It is to attract attention that CS- based polyHIPEs have been slightly studied. Within the few works reported, Miras et al. [41] demonstrated that the HIPE method allows obtaining CS-polyHIPEs with well-defined macroporous structure by using genepin as a natural crosslinker. Then, Zhao et al. [42] synthesized monolithic polyHIPEs of chitosan-g-polyacrylamide for the adsorption of the methylene blue dye. In other work, Zhu et al. [35] reported a novel macroporous magnetic chitosan-g-poly(acrylic acid) hydrogel fabricated from the Pickering HIPE template stabilized by modified Fe_3_O_4_ NPs. This material was satisfactorily used for the adsorption of Cd^2+^ and Pb^2+^. Furthermore, chitosan-g-poly(2-acrylamide-2-methylpropanesulfonic acid) (CS-g-AMPS) porous adsorbent was prepared by grafting the AMPS onto the CS in the Fe_3_O_4_ stabilized Pickering HIPEs and used for the adsorptive removal of the antibiotics tetracycline and chlortetracycline [38].

In this work, iron oxide–chitosan macroporous nanocomposite hydrogels were prepared via Pickering high internal phase emulsion templating and investigated as heterogeneous catalysts for the electro-Fenton degradation of ciprofloxacin in water. The synthesized monoliths were characterized in terms of porous morphology, iron content, crosslinking degree, surface area, and swelling behavior in order to establish the relationship between structure and catalytic performance. Hydroxyl radical generation was also evaluated, and the degradation efficiency of the heterogeneous electro-Fenton system was compared with that of direct anodic oxidation under similar conditions. This study provides new insight into the use of biodegradable chitosan-based macroporous nanocomposite hydrogels as functional catalytic materials for sustainable antibiotic removal from aqueous media.

## 2. Results and Discussion

### 2.1. Stabilization and Structural Morphology of Oil-in-Water Pickering HIPEs

HIPEs having an internal phase (tetradecane) of 80 vol% were prepared with amounts of the triblock surfactant Pluronic^®^ F127 and Fe_3_O_4_ NPs of 0, 2 and 6 wt% and 0.5, 1 and 2 wt% with respect to the continuous phase, respectively. The surfactant Pluronic^®^ F127 is a polypropylene glycol/polyethylene glycol triblock copolymer with a hydrophilic/hydrophobic balance (HLB) of 23 that is commonly used to stabilize oil-in-water HIPEs [28,36]. In addition, Fe_3_O_4_ NPs were chosen for this study because they have been widely used as a stabilizer agent in Pickering HIPEs stabilization and as Fenton catalysts [28,39]. The continuous phase was the chitosan solution, accounting for 20 vol%. The emulsions were named as HIPE-X-Y, where X and Y were the amount of Pluronic F127 and Fe_3_O_4_ NPs, respectively. HIPEs-0-Y (Y = 0.5, 1, 2 wt%) were not stable and presented phase separation (Figure S1B). On the other hand, HIPEs-2-Y (Y = 0.5, 1, 2 wt%) and HIPEs-6-Y (Y = 0.5, 1, 2 wt%) were stable for more than 2 days (Table 1) and had a black gel aspect (Figure S1A).

**Figure 1:**
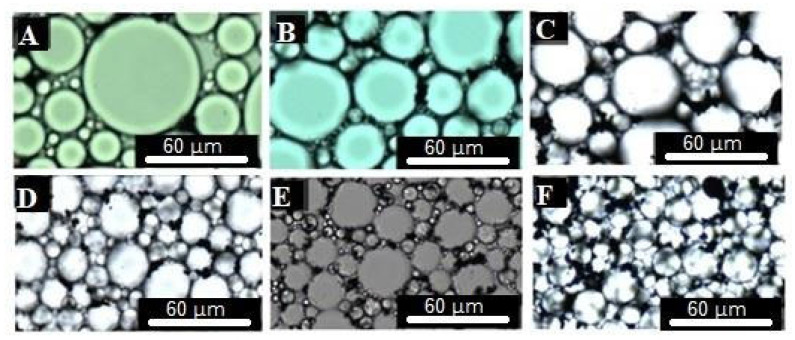
Optical micrographs of (**A**) HIPE-2-0.5, (**B**) HIPE-2-1, (**C**) HIPE-2-2, (**D**) HIPE-6-0.5, (**E**) HIPE-6-1 and (**F**) HIPE-6-2.

The structural morphology of stable emulsions consisted of close-packed polyhedral droplets separated by a thin film of continuous phase, as was observed by optical microscopy (Figure 1). The droplet diameter (D_g_) decreased as the amount of surfactant and Fe_3_O_4_ NPs increased (Table 1). Emulsion stability can be affected by the phenomena of coalescence and Ostwald ripening; hence, the droplet diameter can increase when the emulsion presents low stability [43,44]. Several works [28,29,30,45,46] reported that NPs/surfactants hybrids have been synergistically used to enhance emulsion stability because they can form an effective adsorption barrier at the oil-in-water interface that prevents the phenomena of coalescence and Ostwald ripening. Thus, disperse phase droplets with a more compact conformation can be obtained when the amount of surfactant and Fe_3_O_4_ NPs increases, which could explain the results obtained here.

### 2.2. Porous Structure of the Nanocomposite Chitosan PolyHIPEs

Glutaraldehyde (GA) has been widely used as a crosslinking agent to stabilize CS-based catalysts, as raw CS lacks sufficient mechanical strength and is susceptible to degradation under prolonged exposure to •OH radicals [20,47]. Therefore, crosslinked CS based catalysts have been developed with improved degradation efficiency of organic contaminants. Taking this into account, GA was used as a crosslinking agent to obtain the polyHIPE nanocomposites, as is explained in Section 4. After lyophilizing, PHIPE-2-0.5, PHIPE-2-1 and PHIPE-6-0.5 crumbled, while PHIPE-2-2, PHIPE-6-1 and PHIPE-6-2 had shrinkage (ca. 39 vol %), maintaining the shape of the vessel where the precursor emulsions were prepared (Figure S1C,D and Table 1). All monoliths had crosslinking degrees (C) greater than 86%, determined gravimetrically by Equation (1) described in Section 4 (Table 1).

**Figure 2:**
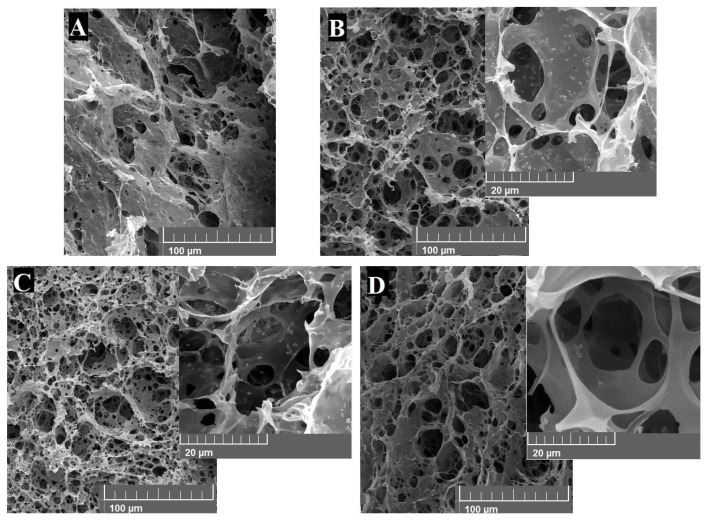
FESEM micrographs at different magnifications of (**A**) image represents the structure of PHIPE-2-0.5, PHIPE-2-1 and PHIPE-6-0.5, (**B**) PHIPE-2-2, (**C**) PHIPE-6-2 and (**D**) PHIPE-6-1 at different magnifications.

Monoliths were observed by field emission scanning electron microscopy (FESEM). As was expected, the porous structure of PHIPE-2-0.5, PHIPE-2-1 and PHIPE-6-0.5 collapsed (Figure 2A). These results show that their precursor emulsions were not stable enough during the crosslinking process. As it was mentioned before, more stable emulsions can be obtained by increasing the amount of surfactant and Fe_3_O_4_ NPs. Therefore, the porous structure of PHIPE-2-2, PHIPE-6-1 and PHIPE-6-2 consisted of a macroporous network highly interconnected through narrow pore windows (D_pw_) that resembled the structural morphology of the emulsions used as templates (Figure 1 and Figure 2). In addition, it was observed that Fe_3_O_4_ NPs were deposited onto the porous surface of the monoliths (insets Figure 2). As well as D_g_, the pore diameter (D_p_) also decreased as the amount of surfactant and Fe_3_O_4_ NPs increased (Table 1).

The equation proposed by Pulko and Krajnc [22] (Equation (2)) was used to determine the degree of openness (O) of the macroporous monoliths. PolyHIPEs presented higher O when the amount of surfactant is increased, which is in agreement with previous works [28,29,36,45,48]. At a higher surfactant amount, the layer of the continuous phase between internal phase droplets thins and begins to retract at points where droplets touch each other, leading to a bigger opening in the cell wall [28,29,36,45,48]. In water treatment applications, polyHIPEs with highly interconnected porosity and high O values are advantageous because the contaminated water can easily flow through the pores and come into contact with the porous surface where NPs are embedded, which enhances the material performance [28,49].

### 2.3. Selection and Characterization of the Electro-Fenton Catalyst

In the heterogeneous electro-Fenton process, the iron catalyst can be supported on solid materials with different structural configurations [11,28,39]. Since the catalytic process takes place on the catalyst surface, it must be accessible to the reactants. Therefore, a desirable property needed for the supporting materials for holding the iron catalysts for the electro-Fenton reaction can be their interconnected porous structure that allows the contaminated water (containing the organic pollutant and H_2_O_2_ at acid pH) to flow through the porous structure and come into contact with the iron catalyst deposited onto the porous surface. In this case, PHIPE-6-2 was chosen as an electro-Fenton catalyst mainly due to its well-defined porous structure with the highest O value (Figure 2C and Table 1) and because bare Fe_3_O_4_ NPs are embedded onto its porous surface.

**Figure 3:**
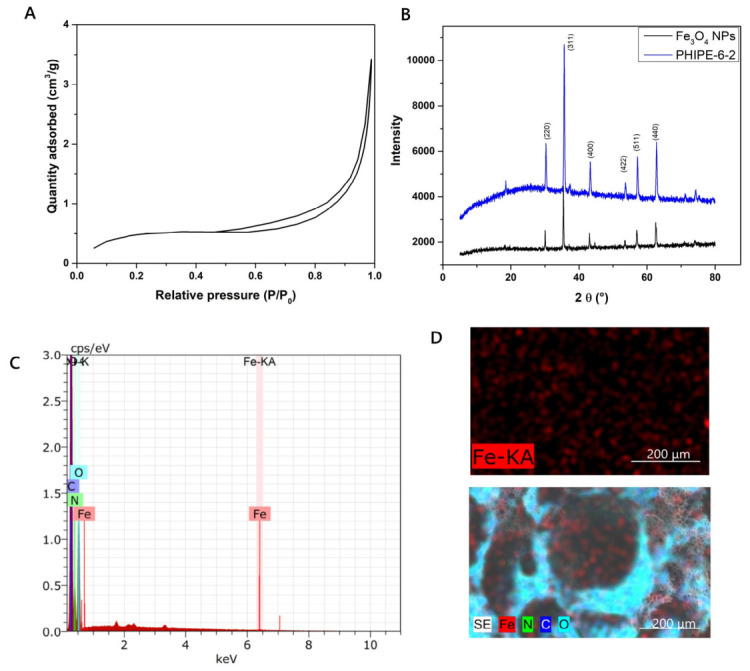
(**A**) Nitrogen adsorption/desorption isotherm, (**B**) XRD patterns of bare Fe_3_O_4_ NPs and PHIPE-6-2, (**C**) spectra EDS and (**D**) elemental mapping of PHIPE-6-2.

Information about the porosity of PHIPE-6-2 was obtained by the nitrogen adsorption/desorption isotherm. The monolith presented an isotherm type II for macroporous materials (according to IUPAC classification) with a narrow hysteresis loop and small adsorption at low pressure (Figure 3A) [50]. The BET analysis showed a surface area of 1.77 m^2^ g^−1^, which is typical of polyHIPE materials [28,31,36].

Furthermore, the monolith was analyzed by energy-dispersive X-ray spectroscopy (EDS) and X-ray diffraction (XRD) to confirm the presence of the Fe_3_O_4_ NPs (Figure 3) and by inductively coupled plasma-atomic-optical emission spectrometry (ICP-OES) to determine the content of the Fe element. The spectra EDS and elemental mapping exposed the presence of the Fe element on the porous surface of the monolith (Figure 3C,D). The XRD pattern (Figure 3B) presents the representative diffraction peaks of (220), (311), (400), (511), and (440) planes of spinel-type iron oxides based on the standard XRD pattern card JCPDS No. 19-0629 [28,29]. Although these results are consistent with Fe_3_O_4_ NPs, it is acknowledged that XRD alone does not allow unambiguous differentiation between Fe_3_O_4_ and maghemite (γ-Fe_2_O_3_) [51]. Magnetite nanoparticles were incorporated prior to HIPE templating, and previous studies have shown that the polymerization and curing processes involved in polyHIPE formation do not induce significant phase transformations under comparable conditions [28,39]. Although the XRD pattern is consistent with magnetite (Fe_3_O_4_), it is acknowledged that XRD alone does not allow unambiguous differentiation between magnetite and maghemite (γ-Fe_2_O_3_), particularly considering that partial oxidation of Fe_3_O_4_ may occur during air exposure, functionalization, aqueous processing, or electro-Fenton operation [51]. Therefore, the material is hereafter referred to as iron oxide NPs when discussing structural characterization and catalytic application.

These results highlight the critical role of emulsion stability, achieved through the synergistic interaction between the surfactant and nanoparticles, in determining the final porous architecture of the monoliths, as well as the effective incorporation and distribution of iron oxides NPs on the pore surface. In this context, obtaining polyHIPE nanocomposites with bare nanoparticles exposed on the porous framework is particularly important for catalytic applications [28,39]. The direct exposure of iron nanoparticles ensures their availability as active sites for the Fenton reaction, avoiding the limitations associated with particle encapsulation or inaccessibility within the polymer matrix. Moreover, the combination of a hydrophilic chitosan-based network and a highly interconnected macroporous structure can promote efficient transport of aqueous pollutants and facilitate their interaction with the catalytic sites [28,39]. This structural design can enhance mass transfer and maximize the utilization of reactive species, ultimately improving catalytic performance. In contrast, systems stabilized solely by nanoparticles often require surface functionalization to achieve suitable interfacial properties and may result in closed-cell structures that limit fluid permeability and reduce the accessibility of active sites [52]. Therefore, the development of polyHIPE nanocomposites with accessible, non-functionalized nanoparticles and open porous architecture represents a key advantage for efficient heterogeneous electro-Fenton catalysis.

In addition, the swelling behavior of the PHIPE-6-2 monolith was assessed by gravimetric and volumetric methods under acidic aqueous conditions (pH 3, 25 °C). The material exhibited rapid water uptake, reaching maximum swelling values of 142.1 ± 2.3% (% S) and 39.3 ± 1.2% (% S_v_) within 15 min, followed by stabilization after ~30 min, indicating equilibrium. The full swelling kinetics curve is presented in Figure S2 in the Supporting Information, allowing a more detailed assessment of the material’s water uptake behavior. The limited and well-controlled swelling response reflects the high crosslinking density of the CS network with GA, which restricts polymer chain relaxation and suppresses excessive hydration [53,54]. This behavior evidences the formation of a mechanically stable and dimensionally preserved macroporous architecture, in agreement with the crosslinking degree reported in Table 1. In contrast to non-crosslinked systems, which typically undergo structural collapse upon hydration [53,54], the present material maintains its integrity under acidic aqueous conditions, supporting its suitability for catalytic applications.

### 2.4. CIP Degradation by Heterogeneous Electro-Fenton Process and Anodic Oxidation

#### 2.4.1. Electrogeneration of Hydrogen Peroxide (H_2_O_2_)

The electrogeneration of hydrogen peroxide (H_2_O_2_) is a key step in the electro-Fenton process, as it enables the controlled production of the oxidant prior to its use in the reaction system. This approach eliminates the need for external addition of H_2_O_2_, reducing safety concerns associated with storage and handling while minimizing excess reagent consumption [9,10,18]. In addition, the continuous supply of electrogenerated H_2_O_2_ allows its effective interaction with iron active sites, promoting the formation of hydroxyl radicals (•OH). Therefore, evaluating H_2_O_2_ production under different operating conditions is essential to understand its influence on •OH generation and, ultimately, on the performance of the heterogeneous electro-Fenton system developed in this work.

To this end, the effect of the applied current density on H_2_O_2_ generation was evaluated in a 50 mL electrochemical cell operated at 25 °C, using a Ti/Pt anode and a carbon cloth–Teflon air diffusion cathode, both with a surface area of 3 cm^2^. Dry air was continuously supplied to the cathode at a flow rate of 1 L min^−1^ as the oxygen source, providing a cost-effective alternative to pure O_2_ while maintaining efficient H_2_O_2_ production, as reported in previous studies [55,56,57]. A 0.05 M Na_2_SO_4_ solution at pH 3 was used as the supporting electrolyte (see Section 4 for further details). H_2_O_2_ was electrogenerated via the two-electron reduction in dissolved oxygen at the cathode [55,56], and the effect of current density (50, 70, and 100 mA cm^−2^) was investigated. The concentration of electrogenerated H_2_O_2_ was determined by UV–Vis spectroscopy based on the formation of a titanium (IV) peroxo-complex (TiO_2_·H_2_O_2_), which exhibits a characteristic absorption at 404 nm [57,58]. In acidic medium, H_2_O_2_ reacts with Ti (IV) species to form a yellow complex, whose absorbance intensity is directly proportional to the H_2_O_2_ concentration. Quantification was carried out using a calibration curve constructed from standard H_2_O_2_ solutions; the corresponding plot and linear regression are provided in the Supplemental Material (Figure S3).

**Figure 4:**
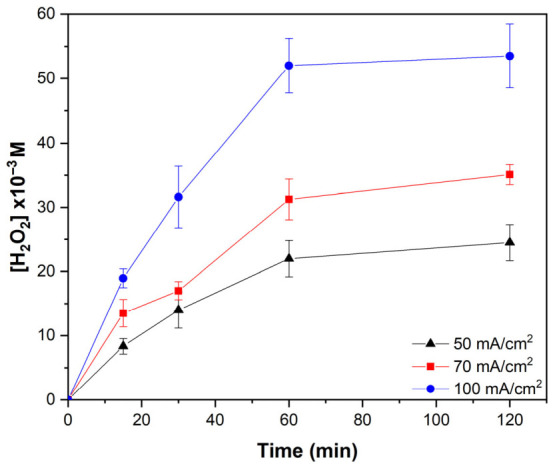
Evolution of electrogenerated hydrogen peroxide (H_2_O_2_) concentration as a function of time at different applied current densities (50, 70, and 100 mA cm^−2^). Error bars correspond to the standard deviation of triplicate measurements.

The results (Figure 4) show that increasing the applied current density led to higher H_2_O_2_ concentrations, with the maximum production observed at 100 mA cm^−2^ due to the greater electron flux available for oxygen reduction. However, although the initial production was higher, the accumulation of H_2_O_2_ decreased more rapidly over time compared with 50 and 70 mA cm^−2^. This behavior can be attributed to the increase in cell potential at higher current densities, which promotes parasitic cathodic reactions such as the further reduction of H_2_O_2_ to water. In addition, part of the generated H_2_O_2_ may decompose at the anode, forming hydroperoxyl radicals (HO_2_•) or undergo complete decomposition into water and oxygen, as reported by Martínez-Huitle et al. [59].

H_2_O_2_ production in air diffusion cathodes is known to depend on several operational parameters, including cathode material, electrode area, applied current density, solution pH, and airflow rate [57]. The trends observed in this study are consistent with those reported in the literature, confirming that higher current densities enhance H_2_O_2_ generation but also intensify side reactions and energy consumption [57]. Based on these considerations, a current density of 70 mA cm^−2^ was selected for subsequent experiments, as it provides a suitable balance between H_2_O_2_ generation efficiency, stability, and energy demand.

#### 2.4.2. Hydroxyl Radical Generation

The degradation of CIP was comparatively evaluated using two independent oxidation systems: direct anodic oxidation and a heterogeneous electro-Fenton process employing iron oxide–chitosan monoliths as catalysts. Prior to assessing the catalytic performance of both systems, the generation of hydroxyl radicals (•OH) was investigated.

For anodic oxidation, experiments were conducted using the same electrochemical cell (50 mL of operational volume) and conditions mentioned before (see Section 4 for further details). In this case, the cathode was bubbled with a N_2_ flow (instead of an air flow) of 0.96-1Lm^−1^ at 35 psi at all times during the essay. The N_2_ flow was used to avoid the generation of H_2_O_2_ and to allow only the production of the hydroxyl radical through H_2_O oxidation [60]. In contrast, the heterogeneous electro-Fenton process was carried out in a packed column containing the synthesized iron oxide–chitosan monoliths (7 g), where electrogenerated H_2_O_2_ solution (50 mL, 35 ± 2 mM) was directly supplied and activated at the iron active sites to generate •OH. Before the oxidation experiments, the monoliths were pretreated by washing with electrogenerated H_2_O_2_ for 30 min in order to remove residual non-crosslinked chitosan that could interfere with the •OH generation. It is important to note that the comparison between both systems is presented here as a practical performance reference under representative conditions, rather than a strictly equivalent evaluation under identical reactor configurations and hydrodynamic conditions.

**Figure 5:**
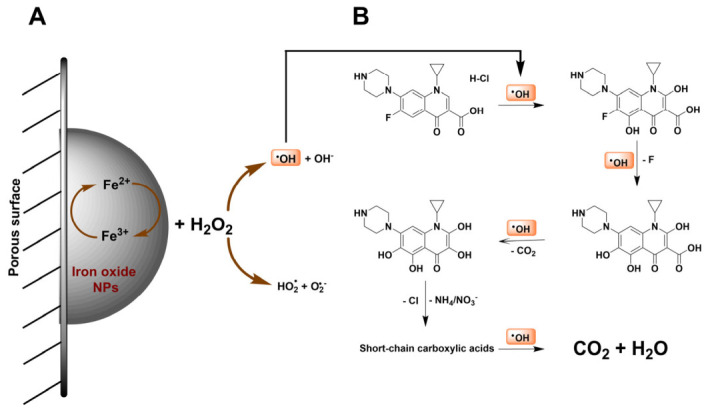
Schematic representation of (**A**) •OH radical generation via the activation of electrogenerated H_2_O_2_ through the Fe^2+^/Fe^3+^ redox cycle at iron oxide active sites available on the porous surface of PHIPE-6-2 monoliths, and (**B**) the proposed mechanism for CIP degradation.

According to the well-established literature [61,62], hydroxyl radicals (•OH) are the dominant and most powerful oxidizing species (E^0^ = 2.8 V/SHE) in AOPs, and are primarily responsible for the degradation and mineralization of organic pollutants. In heterogeneous electro-Fenton catalysis, •OH radicals are generated through the catalytic activation of H_2_O_2_ at the iron oxide active sites via the Fe^2+^/Fe^3+^ redox cycle, a mechanism widely reported in the literature [62]. Therefore, Figure 5A shows a schematic representation of the proposed mechanism for •OH generation. In this process, H_2_O_2_ reacts with surface-bound Fe^2+^ species to produce •OH and Fe^3+^, which is subsequently reduced back to Fe^2+^, sustaining the catalytic cycle. Importantly, the highly interconnected macroporous structure of the synthesized material ensures that these iron oxide active sites are readily accessible, promoting efficient mass transport and enhancing the interaction between H_2_O_2_ and the catalytic surface, which ultimately favors •OH generation. In the case of anodic oxidation, it has been reported that •OH are generated directly at the anode surface via water oxidation, forming adsorbed hydroxyl radicals (•OH__ads_) that can either react with organic pollutants or recombine to form oxygen [62].

Although other reactive oxygen species (ROS), such as hydroperoxyl (HO_2_•) and superoxide (O_2_•^−^) radicals, may also be formed, their contribution is generally secondary due to their lower oxidation potential [61,62]. Therefore, the quantitative determination of •OH provides a direct and reliable descriptor of the oxidative capacity of the systems. This approach enables a meaningful comparison between both oxidation processes and allows the catalytic efficiency of the synthesized material to be evaluated based on its ability to promote H_2_O_2_ activation and enhance •OH generation.

**Figure 6:**
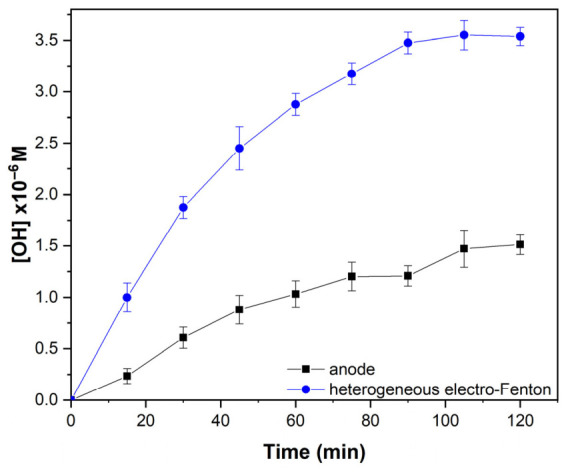
Hydroxyl radical (•OH) generation as a function of time in anodic oxidation and heterogeneous electro-Fenton systems. Anodic oxidation setup operated in a 50 mL electrochemical cell under continuous N_2_ flow (0.96–1 L min^−1^, 35 psi). Heterogeneous electro-Fenton system consisting of a packed column containing PHIPE-6-2 monoliths (7 g), where electrogenerated H_2_O_2_ (50 mL, 35 ± 2 mM) is supplied and catalytically activated at iron active sites to produce •OH radicals; monoliths were pretreated with H_2_O_2_ prior to use. Error bars correspond to the standard deviation of triplicate measurements.

Based on this rationale, the evolution of •OH concentration was monitored and is presented in Figure 6 for both anodic oxidation and heterogeneous electro-Fenton systems. The concentration of •OH was determined using fluorescence spectroscopy with coumarin as a molecular probe, which reacts with •OH to form highly fluorescent 7-hydroxycoumarin [63]. The fluorescence intensity, measured at an excitation wavelength of 350 nm and an emission wavelength of 450 nm, was directly correlated to •OH concentration using a calibration curve constructed with standard solutions of 7-hydroxycoumarin. The corresponding calibration plot is provided in the Supporting Information (Figure S4). As shown in Figure 6, in the case of anodic oxidation, •OH concentrations of approximately 1.5 µM were reached after 100–120 min of reaction. Beyond this period, a significant decrease in the production rate was observed, which can be attributed to the partial passivation of the electrode surface [64] as well as to limitations associated with the quantification method [63]. Similar trends have been reported in other anodic oxidation systems [64,65].

In contrast, under heterogeneous electro-Fenton conditions, using electrogenerated H_2_O_2_ at 70 mA cm^−2^, a higher •OH concentration of up to ~3.5 µM was achieved. This result confirms that the synthesized material provides the necessary catalytic sites to promote the heterogeneous electro-Fenton reaction and highlights its potential application for ciprofloxacin degradation.

It should be noted that, in the presence of both H_2_O_2_ and •OH radicals and in the absence of other oxidizable species, secondary reactions may occur between these oxidants, leading to the formation of hydroperoxyl radicals (HO_2_•) or other less reactive species, as described by Pignatello et al. [8]. These side reactions can influence the effective concentration of •OH and should be considered when interpreting the results.

Importantly, after the heterogeneous electro-Fenton process, negligible total organic carbon (TOC) contribution from the monoliths was detected in water, suggesting that the material maintained its structural integrity and did not undergo significant degradation despite prolonged exposure to highly reactive •OH radicals. This behavior can be attributed to the effective crosslinking of chitosan with GA, which enhances the chemical stability of the matrix under oxidative conditions [18,21,53,54].

#### 2.4.3. CIP Degradation

**Figure 7:**
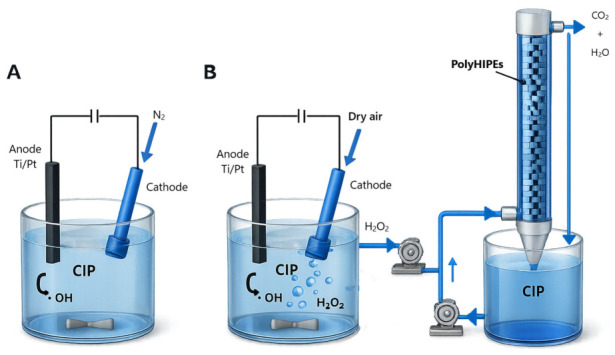
Schematic representation of the lab-scale oxidation systems used for ciprofloxacin (CIP) degradation. (**A**) Anodic oxidation setup, where the electrochemical cell (50 mL of CIP solution at 100 ppm) was operated under N_2_ flow (0.96–1 L min^−1^, 35 psi). (**B**) Heterogeneous electro-Fenton system consisting of a packed column containing iron oxide–chitosan monoliths (polyHIPEs) (7 g), 50 mL of a CIP solution (200 ppm) was mixed with 50 mL of electrogenerated H_2_O_2_ (35 mM), resulting in a final CIP concentration of 100 ppm and ensuring comparable initial conditions between both systems. All tests were carried out at pH 3 and 25 °C. The schematic illustration was generated using ChatGPT 5.3 Go, based on the experimental setup.

Once the successful generation of hydroxyl radicals (•OH) was confirmed, the catalytic performance of the synthesized porous materials was evaluated and compared with anodic oxidation for CIP degradation. Experiments were conducted under conditions similar to those used for •OH generation. For anodic oxidation, 100 mL of a CIP solution (100 ppm) was introduced into the electrochemical cell. In the heterogeneous electro-Fenton system, 50 mL of a CIP solution (200 ppm) was mixed with 50 mL of electrogenerated H_2_O_2_ (35 mM), resulting in a final CIP concentration of 100 ppm and ensuring comparable initial conditions between both systems. All tests were carried out at pH 3 and 25 °C. The comparison between both systems provides a useful performance reference, allowing the relative effectiveness of each oxidation pathway to be assessed and offering insight into the role of the porous catalytic material in enhancing the electro-Fenton process. A schematic representation of the lab-scale oxidation systems used for CIP degradation is given in Figure 7 (see Section 4 for further details).

**Figure 8:**
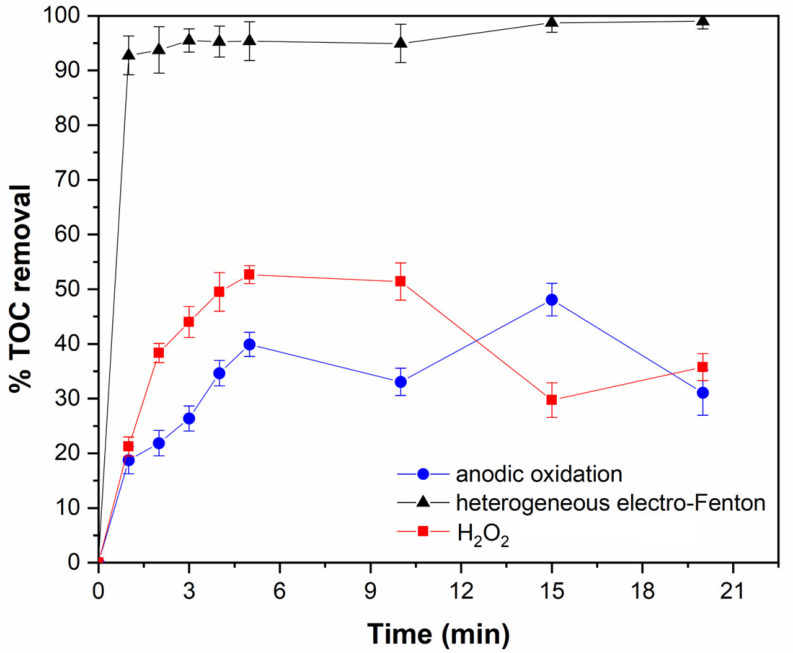
Total organic carbon (TOC) removal efficiencies for ciprofloxacin (CIP) degradation as a function of reaction time under anodic oxidation, heterogeneous electro-Fenton using PHIPE-6-2 monoliths as catalyst, and control conditions with electrogenerated H_2_O_2_ in the absence of a catalyst. Error bars correspond to the standard deviation of triplicate measurements.

Figure 8 presents the TOC removal efficiencies obtained for both anodic oxidation and heterogeneous electro-Fenton systems. In the case of anodic oxidation, a TOC removal of approximately 32% was achieved after 20 min of reaction. This moderate degradation efficiency can be attributed to the low generation (1.5 µM) and non-selective nature of hydroxyl radicals, which may also react with H_2_O_2_ or other species present in the system, as previously discussed.

In contrast, the heterogeneous electro-Fenton process exhibited significantly enhanced performance. Under these conditions, where •OH concentrations of up to ~3.5 µM were achieved, nearly 95% TOC removal was obtained within the first 2 min of reaction, reaching complete mineralization after 15 min. Based on these results, a plausible degradation mechanism can be proposed and illustrated in Figure 5, in agreement with pathways reported in the literature [61]. The process is initiated by the catalytic activation of H_2_O_2_ at the iron oxide active sites via the Fe^2+^/Fe^3+^ redox cycle, leading to the generation of highly reactive •OH radicals. These radicals rapidly attack the ciprofloxacin molecule, primarily targeting the heterocyclic ring, followed by a sequence of oxidation steps including hydroxylation, defluorination, decarboxylation, and progressive ring opening. The resulting intermediates are further oxidized into short-chain carboxylic acids and ultimately mineralized into CO_2_ and H_2_O.

Importantly, the porous architecture of the monolith ensures that the iron oxide active sites are highly accessible, promoting continuous H_2_O_2_ activation and sustained •OH production. This structural feature enhances the interaction between reactants and catalytic sites, accelerating both intermediate transformation and overall mineralization.

**Figure 9:**
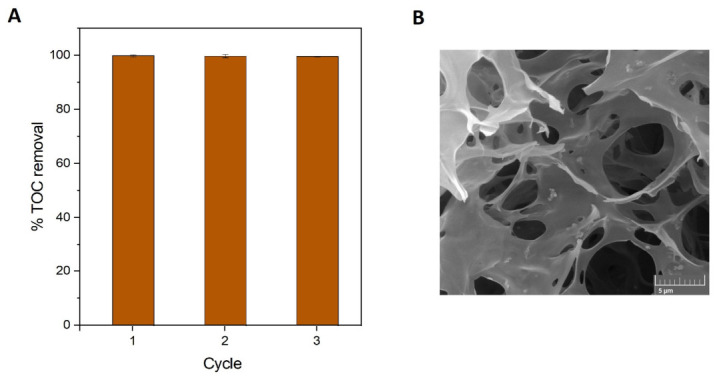
Reusability and structural stability of iron oxide–chitosan monoliths. (**A**) TOC removal efficiencies for CIP degradation over three consecutive cycles. (**B**) FSEM micrographs of the monoliths after three CIP degradation cycles. Error bars correspond to the standard deviation of triplicate measurements.

To further elucidate the catalytic role of the monolith, additional control experiments were conducted. In particular, the CIP degradation in the presence of electrogenerated H_2_O_2_ and in the absence of the catalyst was evaluated. The results showed that H_2_O_2_ alone led to significantly lower degradation and mineralization efficiencies, with only approximately 35% TOC removal achieved after 20 min of reaction. This limited performance can be attributed to the relatively low oxidizing power of H_2_O_2_ in the absence of catalytic activation [62], underscoring the importance of iron active sites for the efficient generation of highly reactive •OH radicals and CIP degradation. Furthermore, the monoliths were reused for three consecutive cycles without significant loss of catalytic performance, maintaining comparable TOC removal efficiencies in each cycle (Figure 9A). Importantly, no noticeable changes in their macroporous structure or mechanical integrity were observed after repeated use, with D_p_ = 17.9 ± 0.6 µm, D_pw_ = 7.1 ± 0.7 µm, and O = 37.6% remaining essentially unchanged, indicating their stability under oxidative conditions (Figure 9B). No evidence of structural collapse or pore coalescence was observed after repeated operation. In addition, ICP-OES results revealed no detectable iron leaching into the solution during the reaction or after reuse, confirming the strong retention of the active phase and supporting the heterogeneous nature of the electro-Fenton process. This stability can be attributed to the strong coordination interactions between iron oxide nanoparticles and the free amino groups of the crosslinked chitosan matrix, as reported in the literature [18].

A comparison with previously reported studies highlights the efficiency of the developed system, see Table S1 in the Supplemental Material. For instance, Gupta and Garg [66] investigated a classical homogeneous Fenton process for the degradation of CIP (initial concentration = 100 ppm), reporting maximum removals of ~70% for CIP and 55% for total organic carbon (TOC) after 45–60 min under optimal conditions ([H_2_O_2_]:[Fe^2+^] = 10, H_2_O_2_ = 14.2 mM, pH 3.0). Similarly, in a homogeneous electro-Fenton system, Yahya et al. [61] reported that although complete transformation of CIP into oxidation intermediates occurred within approximately 10 min, the overall mineralization process was significantly slower, requiring up to 6 h to reach ~94–95% TOC removal at optimal conditions (400 mA and 0.1 mM Fe^2+^). In contrast, the heterogeneous electro-Fenton system developed in this work achieved ~95% TOC removal within 2 min and complete mineralization after 15 min, demonstrating a substantial improvement in mineralization compared to both homogeneous Fenton and electro-Fenton systems.

These differences can be attributed not only to enhanced •OH generation but also to their more effective utilization in the heterogeneous system. The immobilization of iron oxide active sites within a highly interconnected macroporous structure facilitates efficient mass transport and continuous H_2_O_2_ activation, promoting sustained •OH production in the bulk solution. In contrast, homogeneous systems involve dissolved catalytic species, which can lead to parasitic reactions, reduced radical efficiency, and the formation of iron sludge that requires additional separation [8,10,11]. By maintaining the catalytic phase immobilized within the monolithic structure, the present system avoids sludge generation while improving operational simplicity and environmental sustainability.

The results obtained here are also comparable to those reported for heterogeneous Fenton systems, see Table S1 in the Supplemental Material [61,66,67,68,69,70,71,72]. For instance, Diao et al. [67] employed FeS_2_/SiO_2_ microspheres as a heterogeneous Fenton catalyst, achieving nearly complete degradation (100%) of CIP (0.10 mM, ≈33 ppm) after 60 min. In another study, Nie et al. [68] developed hexapod-like pyrite nanosheet clusters via a hydrothermal method, which enabled complete degradation of CIP (20 mg L^−1^) within 10 min at pH 4.0 due to their high adsorption capacity and catalytic activity. It is important to note that these studies were conducted under batch conditions, whereas the system developed in this work operates under continuous flow using a packed-column monolith configuration.

These results demonstrate that the developed system exhibits competitive performance relative to previously reported homogeneous and heterogeneous Fenton-based processes (Table S1), while offering additional advantages associated with continuous operation. The packed-column monolith configuration enables flow-through processing and efficient catalyst recovery, which are desirable features for practical applications. The material also offers sustainability advantages, as it can be safely disposed of at the end of its service life without generating secondary contamination due to the biodegradable nature of the crosslinked chitosan matrix [69]. Altogether, these characteristics demonstrate the potential of the system and pave the way for further studies on long-term stability, hydrodynamics, and scale-up feasibility.

## 3. Conclusions

Iron oxide–chitosan macroporous nanocomposite hydrogels were successfully synthesized via Pickering high internal phase emulsion templating and demonstrated to be efficient heterogeneous catalysts for the electro-Fenton degradation of ciprofloxacin (CIP). The resulting monoliths exhibited a well-defined three-dimensional interconnected porous structure with an average pore size of 18.9 ± 0.7 µm, a window size of 8.1 ± 0.7 µm, and an openness degree of 39.6%, along with a specific surface area of 1.77 m^2^ g^−1^, an iron content of 64.2 mg g^−1^, and a high crosslinking degree (92.1%). Their controlled swelling behavior under acidic aqueous conditions confirmed their hydrogel character and structural stability.

The distribution of iron oxide NPs onto the porous structure ensured the availability of catalytically active sites, while the interconnected macroporous architecture promoted efficient mass transport within the material. As a result, the heterogeneous electro-Fenton system exhibited significantly enhanced performance compared to anodic oxidation, achieving ~95% TOC removal within the first minutes of reaction and complete mineralization within 15 min, whereas anodic oxidation reached only 32% removal after 20 min. This superior performance was attributed to the higher generation of hydroxyl radicals (~3.5 µM) under electro-Fenton conditions, in contrast to the lower radical concentrations obtained during anodic oxidation (~1.5 µM).

Furthermore, the monoliths demonstrated robust structural stability and reusability, maintaining their porosity, integrity, and catalytic efficiency over three cycles. Importantly, negligible contributions of TOC and iron leaching from the material were detected after treatment, indicating high resistance to oxidative degradation and effective retention of the active phase under the studied conditions. In addition, the biodegradable nature of the chitosan matrix offers the potential for safe disposal without generating secondary pollution. These findings suggest that materials may offer a viable approach for advanced wastewater treatment, although further studies are required to assess long-term durability and broader applicability.

## 4. Materials and Methods

### 4.1. Materials

Ciprofloxacin (CIP, HPLC grade, ≥98%), chitosan (CS, 190–310 × 10^3^ gmol^−1^), surfactant Pluronic ^®^F127 (HLB = 23, Mw ≈ 12,600 gmol^−1^), iron (II, III) oxide (Fe_3_O_4_ NPs, nanopowder 50–100 nm diameter particles, ≥97%), tetradecane (Td, ≥99%), glutaraldehyde solution (GA, grade II, 25% in water), coumarin (HPLC grade, ≥99%), titanium (IV) oxysulfate solution (TiOSO_4_, 99%, traces metals basis, 15 wt% in dilute sulfuric acid) and acetic acid (glacial, ACS reagent ≥ 99.7%) were purchased from Sigma-Aldrich (Saint Louis, Mo, USA). Hydrogen peroxide (30.0 wt%), Na_2_SO_4_ (99.6%) and ethanol (96%) were purchased from J.T. Baeker (Center Valley, PA. USA). All chemicals were used as received without further purification. Deionized water was used in all experiments.

### 4.2. Formulation and Characterization of Pickering High Internal Phase Emulsions

The oil-in-water Pickering high internal phase emulsions (HIPEs) were prepared using Td as the disperse phase (accounting for 80 vol% of the emulsion), CS aqueous solution as the continuous phase and Fe_3_O_4_ NPs and the surfactant pluronic F127 as the stabilizer agents. First, 0.1 g chitosan was dissolved in 7 mL of acetic acid aqueous solution (1 vol%). This solution was vortexed at 300 rpm and 25 °C until a homogeneous solution was obtained. The surfactant Pluronic F127 was dissolved in 1 mL of the chitosan solution, and then, Fe_3_O_4_ NPs were added. The chitosan solution/pluronic F127/ Fe_3_O_4_ NPs mixture was vortexed at 1000 rpm for at least 20 min and sonicated for another 20 min to ensure homogeneous mixing. The amount of surfactant pluronic F127 and Fe_3_O_4_ NPs used was 0, 2 and 6 wt% and 0.5, 1 and 2 wt% with respect to the continuous phase, respectively. Subsequently, 4 mL of Td was added dropwise into the solution/pluronic F127/ Fe_3_O_4_ NPs mixture under stirring at 500 rpm. The emulsion was stirred for another 30 min after the addition of the dispersed phase was finished to produce a uniform HIPE. The emulsions were named as HIPE-X-Y, where X and Y referred to the amount of Pluronic F127 and Fe_3_O_4_ NPs, respectively.

Pickering HIPEs were characterized by optical microscopy using an Olympus BX51 microscope (Tokyo, Japan) equipped with a QICAM FAST1394 camera (Surrey, BC, Canada), and images were processed with Linksys 32 software. The mean droplet diameter was determined using ImageJ 1.54r, based on the analysis of 50 individual measurements extracted from the recorded images.

### 4.3. Preparation and Characterization of the Magnetite-Chitosan Macroporous Nanocomposite Hydrogels

**Figure 10:**
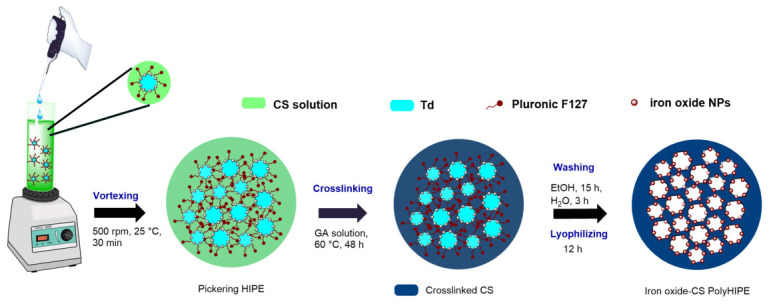
Schematic illustration of the preparation of iron oxide–chitosan polyHIPEs.

The iron oxide–chitosan macroporous nanocomposite materials were labeled as PHIPE-Y-Z, according to the precursor emulsion code. PolyHIPEs were prepared by the addition of 400 µL of GA aqueous solution (5 vol%) into the O/W Pickering HIPE under low-speed stirring (100 rpm) for 3 min. Subsequently, the emulsion was sealed into a glass tube and cured at 60 °C for 48 h. The resulting monolith was washed with ethanol via Soxhlet extraction and water via orbital shaker for 12 and 3 h respectively. Finally, monoliths were lyophilized (IlshinBioBase TFD 8501, Dongducheon-si, Republic of Korea) to remove water for about 12 h. Figure 10 presents a schematic illustration of the preparation of the iron oxide–chitosan monoliths.

The crosslinking percentage of chitosan polyHIPEs, Equation (1), was calculated by an additional Soxhlet extraction for 48 h using a 5 wt% acetic acid solution as extracting solvent [70].

(1) $$Crosslinking\;perecentage\;\left(\%\right)=\left(1-\frac{w}{w_{0}}\;\right)\times 100$$

where w and $w_{0}\;$ are the weights after and before Soxhlet extraction.

After lyophilizing, the macroporous morphology of the chitosan-based polyHIPE monoliths was examined by field emission scanning electron microscopy (FESEM) using a TESCAN MIRA FESEM (Brno, Czech Republic) operated at an accelerating voltage of 10 kV. Prior to imaging, all specimens were sputter-coated with a thin gold layer. Elemental composition was further evaluated by energy-dispersive X-ray spectroscopy (EDS) with a Bruker EDS system (Bremen, Germany). Pore diameters and interconnecting window sizes were quantified using ImageJ 1.54r, based on the average of 50 measurements obtained from SEM micrographs. These parameters were subsequently employed to calculate the degree of openness (O) of the polyHIPE structures, applying the relationship proposed by Pulko and Krajnc (Equation (2)) [22].

(2) $$O=\frac{n\cdot d^{2}}{{\sqrt{3}\;D}^{2}}$$

where n is the average number of pore windows, d is the average pore window diameter, and D is the average pore diameter.

Nitrogen adsorption–desorption measurements were recorded at 77 K using an N_2_ sorption analyzer (ASAP 2020, Micromeritics, Norcross, GA, USA). Prior to the measurements, approximately 0.3 g of each sample was degassed at 50 °C for 8 h under vacuum (≈10 μm Hg) to remove physisorbed species, including water vapor and CO_2_, that could affect the accuracy of the textural characterization. The specific surface area was determined using the Brunauer–Emmett–Teller (BET) model, considering the validity range of the BET equation in the relative pressure interval of 0.05–0.35 (P/P_0_).

The polyHIPE samples were analyzed by attenuated total reflectance Fourier transform infrared spectroscopy (ATR–FTIR) using an ATR accessory coupled to a Bruker Alpha FTIR spectrometer (Bremen, Germany). The crystalline structure of the chitosan monoliths was examined by X-ray diffraction (XRD) employing an Empyrean diffractometer (Almelo, The Netherlands) with CuKα radiation, a step size of 0.02°, and a counting time of 30 s per step. Furthermore, elemental composition was quantified by inductively coupled plasma optical emission spectroscopy (ICP–OES) using a Thermo iCAP 6500 ICP-OES (Cambridge, UK).

The swelling behavior of the monoliths was evaluated using both gravimetric and volumetric approaches. The initial dry mass ($W_{d}$) was recorded, and the initial dimensions were measured using a digital caliper (at least three readings per dimension) to calculate the dry volume ($V_{d}$).

The monoliths (100 mg) were then immersed in 100 mL of distilled water (pH 3) at room temperature under static conditions. At predetermined time intervals, monoliths were removed from the medium, gently blotted with filter paper to remove excess surface water, and weighed and measured to obtain the swollen mass ($W_{s}$) and volume ($V_{s}$). The samples were returned to the swelling medium between measurements until a constant mass/dimensions were reached. All measurements were performed in triplicate.

The gravimetric swelling percentage (S%) was calculated as:

(3) $$S(\%)=\left(\frac{W_{s}-W_{d}}{W_{d}}\right)\times 100$$

The volumetric swelling percentage ($S_{v}\%$) was calculated as:

(4) $$S_{v}(\%)=\left(\frac{V_{s}-V_{d}}{V_{d}}\right)\times 100$$

where $W_{d}$ and $V_{d}$ are the initial dry mass and volume, and $W_{s}$ and $V_{s}$ are the corresponding swollen values at a given time or at equilibrium.

### 4.4. Electrogeneration and Quantification of H_2_O_2_

H_2_O_2_ was electrogenerated in an electrochemical cell consisting of a 180 mL cylindrical reactor with an operational volume of 50 mL, maintained at 25 °C. A Ti/Pt electrode was used as the anode, and a carbon cloth–Teflon air diffusion electrode served as the cathode, both with a surface area of 3 cm^2^. H_2_O_2_ was produced via the two-electron reduction of dissolved oxygen at the cathode by continuously supplying dry, hydrocarbon-free air at a flow rate of 1 L min^−1^ and 35 psi. A 0.05 M Na_2_SO_4_ solution at pH 3 was used as the supporting electrolyte. The effect of current density was evaluated at 50, 70, and 100 mA cm^−2^ using a GWINSTEK GPE-3323 power supply.

The concentration of H_2_O_2_ electrogenerated was determined by UV-Vis spectroscopy (DR6000 Hach spectrometer, Düsseldorf, Germany) from the absorbance of the yellow complex TiO_2_·H_2_O_2_ formed between TiOSO_4_ and the H_2_O_2_ electrogenerated, with a maximum absorbance at 404 nm, as it was described elsewhere [58]. To this end, 40 µL of TiOSO_4_ was added to 1 mL of the electrogenerated H_2_O_2_ solution to form the TiO_2_·H_2_O_2_ complex. Quantification was performed using a calibration curve constructed from standard H_2_O_2_ solutions of known concentration, following the same analytical procedure. The absorbance at 404 nm showed a linear correlation with H_2_O_2_ concentration within the studied range, and the corresponding calibration plot is provided in the Supporting Information (Figure S3). All experiments were carried out in triplicate, and the reported values correspond to the average ± standard deviation.

### 4.5. Ciprofloxacin Degradation by Anodic Oxidation

The degradation of CIP (100 mL, 100 ppm) by anodic oxidation was investigated using the same electrochemical cell and conditions mentioned before. In this case, the cathode was bubbled with a N_2_ flow (instead of an air flow) of 0.96-1 Lm^−1^ at 35 psi at all times during the essay. The N_2_ flow was used to avoid the generation of H_2_O_2_ and to allow only the production of the hydroxyl radical through H_2_O oxidation. Samples (1 mL) were drawn at different times and neutralized with a 0.1 M NaOH solution for quenching the reaction. All experiments were carried out in triplicate. A schematic representation of the lab-scale anodic oxidation process is given in Figure 7A.

### 4.6. Ciprofloxacin Degradation by Heterogeneous Electro-Fenton Process

The iron oxide–chitosan macroporous nanocomposite materials were used as heterogeneous electro-Fenton catalysts for the degradation of the antibiotic ciprofloxacin. The monoliths (7 g) were cut into small cubes of side 5–6 mm and placed into a cylindrical glass column of 300 mm length and 20 mm diameter. First, the monoliths were washed with electrogenerated H_2_O_2_ that was slowly circulated at an addition rate of 0.96-1 Lmin^−1^ through the column for 30 min in order to remove residual non-crosslinked chitosan. Then, the electrogenerated H_2_O_2_ solution (50 mL, 35 ± 2 mM) and a ciprofloxacin solution (50 mL, 100 ppm) were added to the column. At desired times, water samples (1 mL) were drawn and neutralized with a 0.1 M NaOH solution for quenching the reaction. All experiments were carried out in triplicate. A schematic representation of the lab-scale heterogeneous electro-Fenton process is given in Figure 7B.

### 4.7. Determination of Ciprofloxacin Degradation

Total organic carbon (TOC) was used as a measure of the mineralization degree of ciprofloxacin and was determined with a Shimadzu COT-L analyzer (Kyoto, Japan). All measurements were performed in triplicate, and the reported values correspond to the average ± standard deviation.

### 4.8. Determination of ·OH Concentration

The concentration of hydroxyl radical produced by the activation of the electrogenerated H_2_O_2_ on the active sites of iron supported on nanocomposite chitosan polyHIPEs and by the anodic oxidation was determined by fluorescence spectrophotometry (Agilent Technologies Cary Eclypse Fluorescence Spectrophotometer, Mulgrave, VIC, Australia). It has been reported that Coumarin is likely to react with ·OH to produce a highly fluorescent substance, 7-hydroxycoumarin, and the fluorescence intensity is directly proportional to the OH concentration [60]. Therefore, coumarin was used as a molecular probe to form 7-hydroxycoumarin for the quantitative detection of OH concentration. To this end, heterogeneous electro-Fenton and anodic oxidation experiments were carried out with no presence of the ciprofloxacin antibiotic and coumarin was added to the electrolyte support solution at a concentration of 0.2 mM. Samples were taken at different times and measured by fluorescence spectrophotometry at the emission and excitation wavelengths of 450 nm and 350 nm, respectively. Quantification of •OH was performed using a calibration curve constructed from standard solutions of 7-hydroxycoumarin prepared at known concentrations under identical analytical conditions. The fluorescence intensity exhibited a linear correlation with 7-hydroxycoumarin concentration, allowing indirect determination of •OH levels in the systems. The corresponding calibration plot is provided in the Supporting Information (Figure S4). All experiments were conducted in triplicate, and the reported values correspond to the average ± standard deviation, ensuring the reliability of the measurements.

## Figures and Tables

**Table 1:** Structural morphology, stability and % crosslinking of HIPEs and polyHIPEs.

Code X-Y *	HIPE		polyHIPEs				
	Stability (Days)	D_g_ (µm)	Integrity of Monolith	D_p_ (µm)	D_pw_ (µm)	O (%)	C (%)
2-0.5	>2	27.96 ± 0.3	crumbled	-	-	-	93.5
2-1	>2	27.24 ± 0.8	crumbled	-	-	-	96.8
2-2	>2	22.4 ± 0.5	shrinks (ca. 39 vol%)	22.83 ± 0.5	5.2 ± 0.2	19.15	92.9
6-0.5	>7	21.2 ± 0.6	crumbled	-	-	-	86.2
6-1	>7	20.5 ± 0.4	shrinks (ca. 39 vol%)	23.9 ± 1.2	6.2 ± 0.7	29.6	95.9
6-2	>7	19.4 ± 0.3	shrinks (ca. 39 vol%)	18.9 ± 0.7	8.1 ± 0.7	39.6	92.1

* X and Y are the amount of surfactant and Fe_3_O_4_ NPs used in HIPEs formulation; D_g_ is the droplet diameter; D_p_ is the pore diameter; D_pw_ is the pore window; O is the degree of openness; and C is the crosslinking degree.

## Data Availability

Data is contained within the article or Supplementary Material. The original contributions presented in this study are included in the article/Supplementary Material. Further inquiries can be directed to the corresponding authors.

## Supplementary Materials

The following supporting information can be downloaded at: https://www.mdpi.com/article/10.3390/gels12050434/s1, Figure S1: photographs of the emulsion templating process, including (A) stable HIPEs, (B) phase-separated HIPEs, and the resulting monoliths (C) before and (D) after washing and lyophilization. Figure S2: gravimetric (% S) and volumetric (% Sv) swelling behavior of the PHIPE-6-2 monolith as a function of time. Figure S3: calibration curve for electrogenerated hydrogen peroxide (H_2_O_2_) obtained by UV–Vis spectroscopy using the titanium (IV) oxysulfate method (TiO_2_·H_2_O_2_ complex), measured at 404 nm. Figure S4: calibration curve for hydroxyl radical (•OH) determination using coumarin as a fluorescent probe. Table S1 compiles a comprehensive bibliographic review of ciprofloxacin (CIP) degradation through Fenton and electro-Fenton processes, including both homogeneous and heterogeneous systems [61,66,67,68,71,72,73,74].

## Abbreviations

The following abbreviations are used in this manuscript:

AOPs	Advanced oxidation processes
CIP	Ciprofloxacin
CS	Chitosan
DES	Deep eutectic solvent
D_p_	Pore diameter
D_pw_	Window (pore throat) diameter
FESEM	Field emission scanning electron microscopy
GA	Glutaraldehyde
HIPE	High internal phase emulsion
HO_2_•	Hydroperoxyl radical
H_2_O_2_	Hydrogen peroxide
O	Openness degree
PHIPE	Polymerized high internal phase emulsion (monolith)
polyHIPE	Polymerized high internal phase emulsion
TOC	Total organic carbon
UV–Vis	Ultraviolet–visible spectroscopy
λ__em_	Emission wavelength
λ__ex_	Excitation wavelength
% S	Gravimetric swelling
% S_v_	Volumetric swelling
•OH	Hydroxyl radical
ROS	Reactive oxygen species
