# Supplementary material for: Iron Oxide–Chitosan Macroporous Nanocomposite Hydrogels for Efficient Heterogeneous Electro-Fenton Degradation of Ciprofloxacin
Source: Gels. 2026 May 15;12(5):434. doi: 10.3390/gels12050434 (PMC13205368; doi:10.3390/gels12050434)
Supplement: Supplementary file 1 [file gels-12-00434-s001.zip › gels-4263404-supplementary.pdf]

# Iron oxide-Chitosan Macroporous Nanocomposite Hydrogels for Efficient Heterogeneous Electro-Fenton Degradation of Ciprofloxacin

José Benito Pelayo-Vázquez, Daryl Rafael Osuna-Laveaga , José Patricio Peña-Jaramillo, Sergio Gómez-Salazar, Edgar David Moreno-Medrano and María Guadalupe Pérez-García

## Supplemental Material

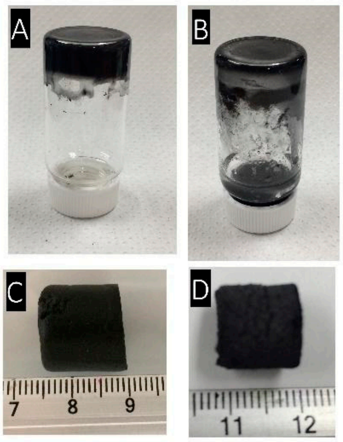


Figure S1. Photographs represent A) stable HIPEs, B) phase separation of HIPEs and monoliths C) before and D) after washing and lyophilizing.

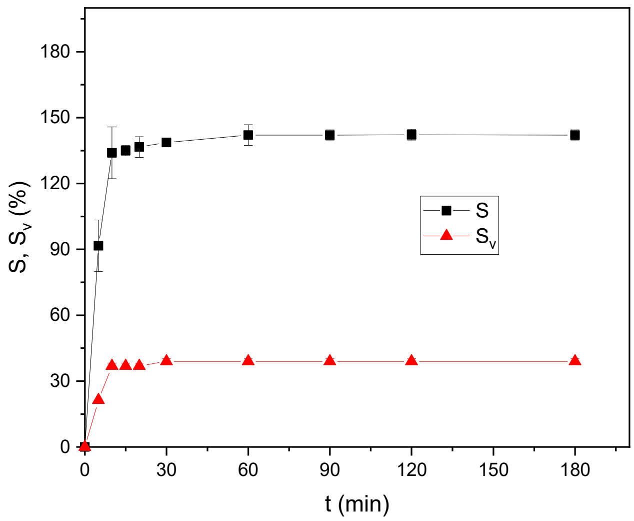


Figure S2. Gravimetric (%S) and volumetric (%$S_v$) swelling behavior of the PHIPE-6-2 monolith as a function of time.

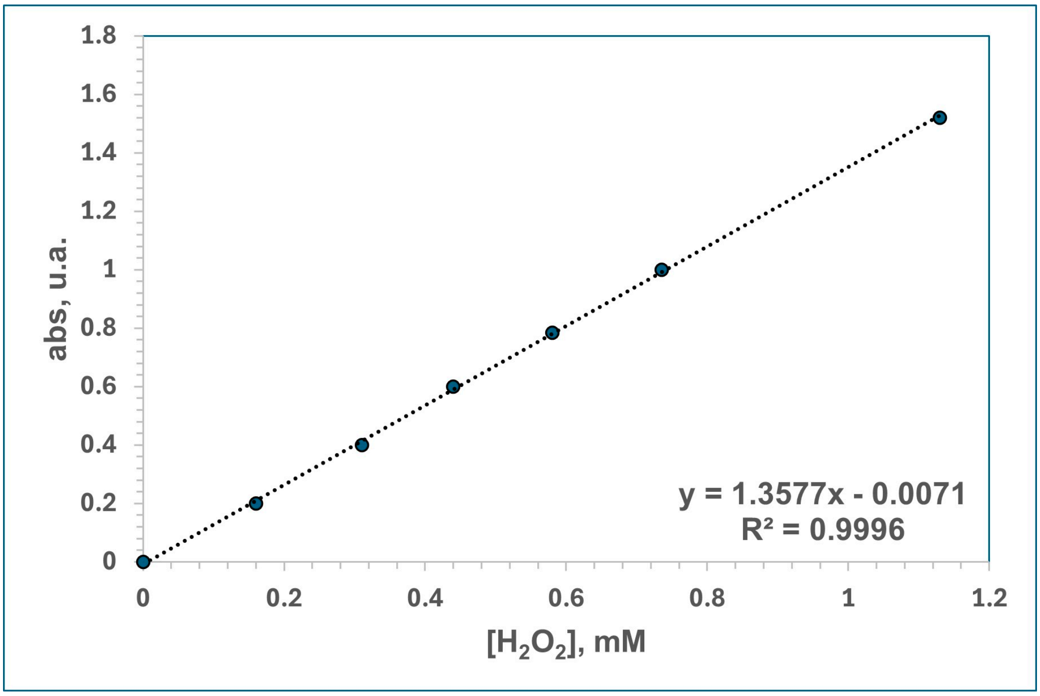


Figure S3. Calibration curve for electrogenerated hydrogen peroxide ($H_2O_2$) obtained by UV–Vis spectroscopy using the titanium(IV) oxysulfate method ($TiO_2 \cdot H_2O_2$ complex), measured at 404 nm.

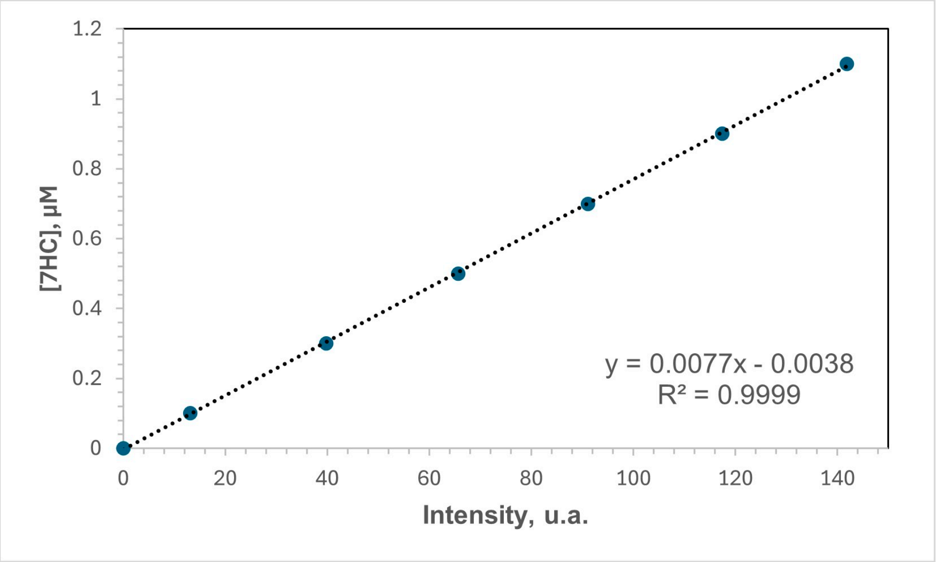


Figure S4. Calibration curve for hydroxyl radical (•OH) determination using coumarin as a fluorescent probe. •OH radicals react with coumarin to form 7-hydroxycoumarin, whose fluorescence intensity (λ_ex = 340 nm, λ_em = 456 nm) is directly proportional to •OH concentration. The resulting linear correlation was used to quantify •OH generated during anodic oxidation and heterogeneous electro-Fenton processes.

**Table S1.** Summary of reported studies on ciprofloxacin (CIP) degradation by Fenton and electro-Fenton processes, including both homogeneous and heterogeneous systems.

| System | COT degradation /time | $[CIP]_0$ / pH | Reference |
|---|---|---|---|
| Homogeneous Fenton | 37.9 %<br>45 min | $[CIP]_0$ = 15 ppm<br>pH 3.5 | [1] |
| Homogeneous Fenton | 55 %<br>60 min | $[CIP]_0$ = 100 ppm<br>pH 3 | [2] |
| Homogeneous electro-Fenton | 95 %<br>6 h | $[CIP]_0$ = 0.15 mM<br>pH 3 | [3] |
| Heterogeneous Fenton<br>$FeS_2/SiO_2$ | 100%<br>60 min | $[CIP]_0$ = 0.10 mM<br>pH 3 | [4] |
| Heterogeneous Fenton<br>Pyrite | 100 %<br>10 min | $[CIP]_0$ = 20 ppm<br>pH 4 | [5] |
| Heterogeneous Fenton<br>Biochars | 90%<br>4 h | $[CIP]_0$ = 10 ppm<br>pH 4 | [6] |
| Photo-Fenton<br>Iron chelation | 93.1%<br>45 min | $[CIP]_0$ = 50ppm<br>pH 3.5 | [7] |
| Heterogeneous Electro-Fenton<br>magnetite | 97.3 %<br>180 min | $[CIP]_0$ = 10 ppm<br>pH 3 | [8] |
| Heterogeneous Electro-Fenton<br>Magnetite-CS PolyHIPE | 95%<br>2min<br>Complete mineralization<br>20 min | $[CIP]_0$ = 100 ppm<br>pH 3 | This work |

# References


1. Giri, A.S.; Golder, A.K. Ciprofloxacin degradation from aqueous solution by Fenton oxidation: Reaction kinetics and degradation mechanisms. *RSC Adv.* **2014**, *4*, 6738–6745.
2. Gupta, A.; Garg, A. Degradation of ciprofloxacin using Fenton's oxidation: Effect of operating parameters, identification of oxidized by-products and toxicity assessment. *Chemosphere* **2018**, *193*, 1181–1188.
3. Yahya, M.S.; Oturan, N.; El Kacemi, K.; El Karbane, M.; Aravindakumar, C.T.; Oturan, M.A. Oxidative degradation study on antimicrobial agent ciprofloxacin by electro-Fenton process: Kinetics and oxidation products. *Chemosphere* **2014**, 117, 447–454.
4. Diao, Z.H.; Xu, X.R.; Jiang, D.; Li, G.; Liu, J.J.; Kong, L.J.; Zuo, L.Z. Enhanced catalytic degradation of ciprofloxacin with $FeS_2/SiO_2$ microspheres as heterogeneous Fenton catalyst: Kinetics, reaction pathways and mechanism. *J. Hazard. Mater.* **2017,** *327*, 108–115.
5. Nie, X.; Li, G.; Li, S.; Luo, Y.; Luo, W.; Wan, Q.; An, T. Highly efficient adsorption and catalytic degradation of ciprofloxacin by a novel heterogeneous Fenton catalyst of hexapod-like pyrite nanosheets mineral clusters. *Appl. Catal. B Environ.* **2022**, *300*, 120734.
6. Li, J.; Pan, L.; Yu, G.; Xie, S.; Li, C.; Lai, D.; Li, Z.; You, F.; Wang, Y. The synthesis of heterogeneous Fenton-like catalyst using sewage sludge biochar and its application for ciprofloxacin degradation. *Sci. Total Environ.* **2019**, *654*, 1284–1292.
7. Giri, A.S.; Golder, A.K. Ciprofloxacin degradation in photo-Fenton and photocatalytic processes: Degradation mechanisms and iron chelation. *J. Environ. Sci.* **2019**, *80*, 82–92,
8. Nidheesh, P.V.; Gandhimathi, R.; Velmathi, S.; Sanjini, N.S. Magnetite as a heterogeneous electro-Fenton catalyst for the removal of Rhodamine B from aqueous solution. *RSC Adv.* **2014**, *4*, 5698–5708.
